# Genetic monkeys reveal a new role for a longevity protein in embryonic development

**DOI:** 10.1093/nsr/nwz051

**Published:** 2019-06-14

**Authors:** Yuyu Niu

**Affiliations:** Yunnan Key Laboratory of Primate Biomedicine Research, Institute of Primate Translational Medicine, Kunming University of Science and Technology, China

As it is involved in boosting lifespan in mammals, *SIRT6* has been in the spotlight for years. In mice, *SIRT6* overexpression leads to improved health and lifespan extension [1]. Animals die soon after birth if SIRT-6 is deficient [2]. A recent study in humans showed that *SIRT6* mutation causes abnormal brain development and results in embryonic death [3]. This suggested that *SIRT6* may play a different role in primate development. In a recent study published in *Nature*, a team of Chinese Academy of Sciences scientists generated a *SIRT6*-deficient macaque monkey by clustered regularly interspaced short palindromic repeats-Cas9. The study showed that the gene, as well as its well-known role in aging, has an additional function in embryonic development [4].

One male and three female macaque monkeys that did not express *SIRT6* exhibited whole-body developmental delays *in utero*, and died during gestation or shortly after birth. Via a series of comprehensive analyses, either using monkey tissue or human embryonic stem cells, the authors found that an absence of *SIRT6* caused lower bone density, lower levels of fat, and immature intestines and skeletal muscle in mutant monkeys. These alterations led to developmental delay and death. Notably, the brains of SIRT6 deficient monkeys were smaller compared than those of wild-type monkeys. The immature brains resulted from delayed neuronal maturation and increased numbers of immature neural progenitor cells (NPCs). Next, the authors examined gene expression in the mutants and found that H19, a long non-coding RNA, was one of the most upregulated genes. H19 expression in the brain was at the highest level detected. They further investigated its roles in the brain and its relationship with *SIRT6*. By generating SIRT6-null human embryonic stem cells and then differentiating them into NPCs, it was observed that SIRT6-null NPCs displayed delayed neuronal differentiation and abundant H19 transcripts. The results indicate that *SIRT6* functions as a mediator of primate brain development by repressing the expression of H19 long non-coding RNA in a *trans* manner.

In general, Zhang *et al*. have shown that the SIRT6 protein has a previously unknown role in embryonic development in primates that has not previously been reported in mice. This study highlights the possibility of generating genetically engineered monkeys to uncover the functions of genes. Consistent with previous studies [5], genetic mutant monkeys can show characteristics of conditions that either are difficult to assess or that have not previously been analyzed in mouse models. Although technological and ethical hurdles remain, one may predict that biomedical research with genetically engineered monkey may revolutionize the study of human diseases.
